# Single Molecule Analysis of Laser Localized Interstrand Crosslinks

**DOI:** 10.3389/fgene.2016.00084

**Published:** 2016-05-09

**Authors:** Jing Huang, Himabindu Gali, Manikandan Paramasivam, Parameswary Muniandy, Julia Gichimu, Marina A. Bellani, Michael M. Seidman

**Affiliations:** Laboratory of Molecular Gerontology, National Institute on Aging, National Institutes of HealthBaltimore, MD, USA

**Keywords:** DNA damage, replication, interstrand crosslinks, single molecule, DNA fiber, laser

## Abstract

DNA interstrand crosslinks (ICLs) block unwinding of the double helix, and have always been regarded as major challenges to replication and transcription. Compounds that form these lesions are very toxic and are frequently used in cancer chemotherapy. We have developed two strategies, both based on immunofluorescence (IF), for studying cellular responses to ICLs. The basis of each is psoralen, a photoactive (by long wave ultraviolet light, UVA) DNA crosslinking agent, to which we have linked an antigen tag. In the one approach, we have taken advantage of DNA fiber and immuno-quantum dot technologies for visualizing the encounter of replication forks with ICLs induced by exposure to UVA lamps. In the other, psoralen ICLs are introduced into nuclei in live cells in regions of interest defined by a UVA laser. The antigen tag can be displayed by conventional IF, as can the recruitment and accumulation of DNA damage response proteins to the laser localized ICLs. However, substantial difference between the technologies creates considerable uncertainty as to whether conclusions from one approach are applicable to those of the other. In this report, we have employed the fiber/quantum dot methodology to determine lesion density and spacing on individual DNA molecules carrying laser localized ICLs. We have performed the same measurements on DNA fibers with ICLs induced by exposure of psoralen to UVA lamps. Remarkably, we find little difference in the adduct distribution on fibers prepared from cells exposed to the different treatment protocols. Furthermore, there is considerable similarity in patterns of replication in the vicinity of the ICLs introduced by the two techniques.

## Introduction

After several decades of effort by many laboratories, we have a detailed understanding of the cellular biochemistry that removes single strand adducts and repairs single and double strand breaks (DSBs). Imaging strategies, particularly those based on immunofluorescence (IF) microscopy, have been particularly helpful in elucidating these pathways and the factors that drive them. Perhaps the most extensive application of IF has been in the study of DSB repair. Fortuitously, proteins that accumulate in the vicinity of breaks introduced by irradiation, replication fork breakage, or enzymatic cleavage, form foci readily visualized by IF. This feature has been widely exploited and has been instrumental in defining the collective of proteins known as the DNA damage response (DDR), the subject of an enormous, and expanding, literature (Ciccia and Elledge, 2010). The appearance of foci in response to DSBs provides an important experimental tool for studying the complexity of the DDR, the timing of accumulation of specific factors to foci, and the dependence of one factor on another for recruitment. Furthermore, although the breaks are not explicitly detected, the proteins of the DDR foci serve as surrogate markers of their presence (Rothkamm et al., 2015).

An additional, and very powerful, technology introduced by the Bonner lab, demonstrated that exposure to localized laser light could provoke the introduction of DSBs which activated the DDR (Rogakou et al., 1999). Analyses of the early marker of the DDR, the now famous phospho-histone, γ-H2AX, by IF, revealed a stripe of signal corresponding to the line of laser exposure across the nucleus. In effect, the experimentalist created the “focus” with the laser. This has proven to be an immensely popular approach to studying the protein dynamics induced by DSBs. γ-H2AX foci are commonly assumed to be indicators of breaks. However, these are not necessarily quantitative reporters of breaks, and it has been difficult to address fundamental questions regarding DSB density and distribution in the regions of interest (ROI).

The obvious power of these imaging strategies raises the possibility of their use in the study of lesions other than DSBs. However, other forms of DNA damage are not as cooperative. Ultraviolet light introduces covalent adducts that, although they attract repair components of the DDR, do not directly stimulate the formation of discrete foci (see below). Similarly, many other DNA adducts do not induce DDR foci except as they block DNA replication. In those situations the disrupted replication fork is the actual instigator of the DDR. In order to follow the cellular response to the lesion, the problem posed by the absence of foci can be overcome by taking advantage of techniques to create foci. These approaches introduce adducts, in a restricted nuclear region, at a density that is higher than in the surrounding area. The accumulation of responsive proteins to these “artificial” foci can then be monitored. This has been possible with UV photoproducts by masking cells with micropore filters which block the passage of short wave UV. Only light passing through an occasional vertical pore reaches the cell layer. This results in nuclei with patches of photoproducts that can be detected and displayed by IF with photoproduct specific antibodies. Protein recruitment to the localized photoproducts can be monitored as well (Volker et al., 2001). This approach is limited to studies of adducts whose formation is dependent on light that can be blocked by the filters, typically ultraviolet photoproducts.

Analogous to the work with DSBs, it is also possible to use lasers of appropriate wavelength to introduce, in defined ROI in nuclei, adducts that are generated by photochemistry. UV photoproducts can, of course, be formed directly (Dinant et al., 2007), while exposure of DNA to radicals produced by absorption of laser energy by endogenous photosensors can produce oxidative lesions and/or single strand breaks (Lan et al., 2004).

Another DNA modification that is of great current interest is the interstrand crosslink (ICL). Agents that form them are highly toxic and are frequently used in cancer chemotherapy. ICLs present a potent challenge to replication and transcription as they are absolute blocks to unwinding of DNA. Consistent with their linkage of two strands, ICLs are repaired in two cycles of repair. In the first the two strands are uncoupled from one another in a process termed “unhooking” (Dronkert and Kanaar, 2001). This can be done either by endonucleolytic incisions on one strand on either side of an ICL, or by introduction of a nick in one of the strands followed by exonucleolytic digestion past the ICL (Sengerova et al., 2012). In both scenarios the “other strand” retains the resultant single strand adduct, termed the “crosslink remnant.” After gap filling, necessarily involving a lesion bypass step, the crosslink remnant is removed by conventional nucleotide excision repair. Although this general scheme has been appreciated for many years, it has become apparent that there are many different proteins involved, and multiple pathways, most incompletely understood.

The field of ICL repair has been greatly stimulated by the establishment of an *in vitro* system, based on *Xenopus* egg extracts, that supports replication, and repair of a plasmid carrying a site specific ICL (Raschle et al., 2008). The very elegant work from the Walter lab has captured the imagination of the field and framed much of the current discussion. However, while a very productive experimental approach, analyses of ICL repair with plasmid based strategies are much influenced by the particulars of the assays. Furthermore, the events modeled by the plasmid systems represent a subset of those at the genomic level. Constructs that measure repair by relief of transcription suppression, of necessity have strong promoters and thus report features of transcription coupled repair of the ICLs (Hlavin et al., 2010). Plasmids that are templates for replication display events related to repair of ICLs in the context of replication fork encounters (Shen et al., 2009; Knipscheer et al., 2012). In order to parse the complexity of ICL repair in the mammalian genome other approaches are required.

We have developed two strategies, based on fluorescence imaging, for addressing questions about the encounter of replication forks with, and the cellular response to, genomic ICLs. At the heart of our experiments is a crosslinking compound whose adducts can be detected immunologically. This enables display of ICLs, and events associated with them, by various imaging techniques. At the outset of our studies we considered the experimental utility of clinically important crosslinking agents. Although Mitomycin C and *cis*-platinum are well established in cancer chemotherapy, they form a minority of ICLs as a fraction of total adducts (<10%), which are not easily detected (Muniandy et al., 2010).

By contrast, the photoactive psoralens can form a very high frequency of ICLs (Lai et al., 2008). The requirement for photo-activation by long wave ultraviolet light (UVA) allows ICL formation to be controlled as to time and location. Furthermore, although psoralens cannot be imaged directly, they can be linked to detection tags. These are moieties that can be imaged directly, such as fluorescent proteins (Chalfie et al., 1994). Another example would be the FLAG peptide which can be detected by antibodies (Knappik and Pluckthun, 1994), or biotin which is bound by streptavidin (Nerurkar et al., 1984). Appropriately conjugated tags, including biotin and fluorescent molecules, can be participants in coupling reactions such as those employed in click chemistry, which has been applied in many areas of biological research (Kolb et al., 2001). As described below, we synthesized a psoralen linked to digoxigenin, a plant sterol not found in mammalian cells, and widely used as an immunotag (Miller et al., 1993).

In one approach, we combined immuno-quantum dot detection of single molecules with DNA fiber technology (Schwab and Niedzwiedz, 2011). We were able to visualize replication tracts in the vicinity of individual psoralen ICLs generated by photoactivation with a UVA lamp (Huang et al., 2013; Rohleder et al., 2016). In the other development, we used a confocal microscope equipped with a UVA laser to introduce psoralen ICLs into ROI in nuclei of live cells. The localized ICLs induce the recruitment of repair and DDR proteins (see below).

The antigen tag on the psoralen allows display of psoralen ICLs, by IF, in a laser photoactivated ROI. Although very useful for monitoring the repair of psoralen ICLs it is not possible, given the limit of resolution of confocal microscopes, to assess the density of ICLs along a DNA molecule within a ROI. In contrast, the single molecule resolution of the fiber/quantum dot technology readily permits determination of ICL frequency and distribution on an individual DNA strand. Thus, an important feature of our DNA fiber experiments is unavailable in the studies with the laser localized ICLs. As lasers can provide photoactivating light at much higher intensity than UVA lamps, it is possible that there are much higher lesion densities in the one technique than the other. Consequently, it is not certain that results with one light source could be directly compared with those of the other, particularly as regards replication in the laser defined ROIs.

In the experiments reported here, we illustrate applications of laser localization and immuno-quantum dot imaging of ICLs. We have used the latter technology to answer fundamental questions about the frequency and distribution of the laser localized ICLs in the ROI. In addition, we directly address the issue of replication in the vicinity of laser induced ICLs.

## Materials and Methods

### Cells and Reagents

HeLa cells were grown in DMEM supplemented with penicillin and streptomycin and 10% fetal bovine serum. Dig-TMP was prepared by conjugation of trimethylpsoralen (TMP) to digoxigenin as described previously (Thazhathveetil et al., 2007). Briefly, the 4′-chloromethyl derivative of TMP was reacted with 4,7,10-trioxa-1,13-tridecanediamine to give TMP with a soluble glycol amine sidechain. This was reacted with the digoxigenin *N*-hydroxysuccinimide ester to give TMP linked to digoxigenin.

### Laser Localized ICLs

A central cross was marked with a diamond pen on the growth surface of a 35-mm glass bottom culture dish (MatTek^TM^). The plates were also scribed on the side of the dish to provide an orientation mark. Cells were seeded so as to achieve 50–60% confluence at the time of an experiment. They were incubated with the 20 μM Dig-TMP at 37°C for 20 min prior to photoactivation. Localized irradiation was performed using a Nikon Eclipse TE2000 confocal microscope with an SRS NL100 nitrogen laser-pumped dye laser (Photonics Instruments, St. Charles, IL, USA). The 365 nm laser shoots 3-ns pulses with a repetition rate of 10 Hz. The power, measured at the back aperture of the ×60 objective, was 0.7 nW. The laser was directed to deliver pulses to a specified rectangular ROI (3 pixel × 30 pixel, 0.16 μm/pixel) within the nucleus of a cell, visualized with a Plan Fluor ×60/1.25 numerical aperture oil objective. The laser, controlled by Volocity-5 software (PerkinElmer Life Sciences), was oriented by galvanometer-driven beam displacers and fired randomly throughout the region until the entire ROI was exposed. Throughout an experiment, cells were maintained at 37°C, 5% CO_2_, and 80% humidity in an environmental chamber.

### Dig-TMP Photoactivation by UVA Lamp

Cells were incubated with indicated concentrations of Dig-TMP, placed in a Rayonet chamber (Southern New England Ultraviolet Co.), and exposed to 365 nm light (3 J/cm^2^). The temperature in the chamber was maintained at 37°C. The cells were either fixed for immunostaining or returned to the incubator for the specified times.

### Immunostaining

Fixed cells were permeabilized with 0.5% Triton X-100, 1% bovine serum albumin, 100 mM glycine, and 0.2 mg/ml EDTA in PBS on ice for 10 min. The cells were subsequently digested with RNase A in PBS-EDTA (5 mM) solution for 30 min at 37°C. Cells were blocked in 10% goat serum in PBS and 0.01% sodium azide for 1 h at 37°C or overnight at 4°C. For IF staining, cells were incubated with appropriate primary antibody diluted in blocking solution for 1 h at 37°C. After three washes using 0.05% Tween 20 in PBS, cells were incubated with a corresponding fluorescence-tagged secondary antibody [Alexa Fluor goat anti-mouse or Alexa Fluor goat anti-rabbit (Invitrogen)]. After three washes, cells were mounted with ProLong Gold antifade reagent with 4′,6-diamidino-2-phenylindole (Invitrogen). Stained cells were visualized and imaged using a Hamamatsu EM-CCD digital camera attached to the Nikon Eclipse TE2000 confocal microscope.

### DNA Fiber Analysis

Cells were incubated for 24 h with 20 μM CldU. They were then incubated with 20 μM Dig-TMP for 20 min before UVA irradiation in a Rayonet chamber or exposure to the 365 nm laser in ROI in nuclei in cells in the immediate vicinity of the cross. Cells exposed to the UVA lamp were harvested by trypsinization and the cells were placed on a glass slide. Plates with cells containing laser localized ICLs were washed and a drop of trypsin from a drawn out capillary placed on the intersection of the cross. The detached cells were recovered and placed on a glass slide. Cells were then mixed with lysis buffer (0.5% SDS in 200 mM Tris-HCl [pH 7.5], 50 mM EDTA) on the slide. After tilting, the slides were air-dried, fixed in 3:1 methanol/acetic acid, incubated in 2.5 M HCl, neutralized in 0.4 M Tris-HCl (pH 7.5), washed in PBS, and immunostained. Antibodies and dilutions were rat anti-BrdU (CldU), 1:200; Dylight 649 goat anti-rat, 1:100; mouse anti-BrdU (IdU), 1:40 and chicken anti-digoxigenin, 1:200; and Dylight 488 goat anti-mouse, 1:100 and Q dot 655 goat anti-chicken, 1:2,500. Imaging was carried out using a Zeiss Axiovert 200 M microscope with the AxioVision software packages (Zeiss). The quantum dot signal was imaged with a Q dot 655 filter.

## Results

### The Tag

**FIGURE 1:**
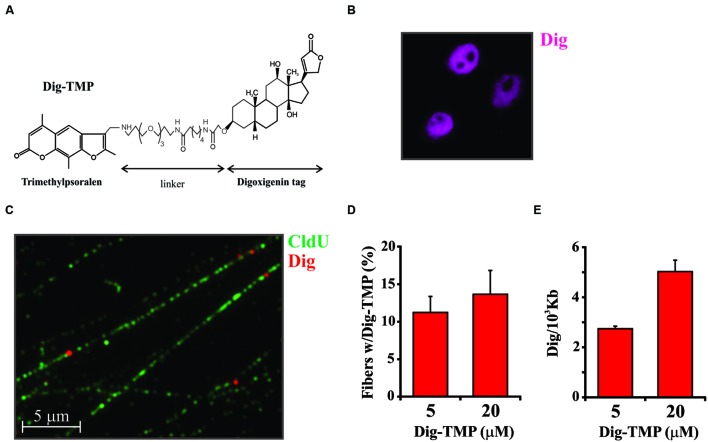
**Distribution of Dig-TMP on DNA fibers from cells after photoactivation by UVA lamp. (A)** The structure of trimethylpsoralen (TMP) linked to Digoxigenin (Dig). **(B)** Cells were incubated with Dig-TMP and exposed to UVA in the Rayonet lamp chamber. They were then immunostained against the Dig tag. **(C)** Cells were prelabeled with CldU, then incubated with Dig-TMP and exposed to the UVA lamp. DNA fibers were prepared and immunostained for CldU (green) and Dig-TMP (red dots). **(D)** Cells were treated with the indicated concentration of Dig-TMP and DNA fibers prepared and stained, and fields photographed as in **(C)**. The fraction of fibers in a field containing a Dig-TMP signal was determined. From cells treated with 5 μM Dig-TMP the number of fibers with a Dig signal and the total fibers scored in individual experiments were: 33 and 297; 25 and 279; 32 and 224. From cells treated with 20 μM Dig-TMP: 39 and 285; 21 and 208; 35 and 206 (total fibers 742, 604). The Chi squared test showed no significant difference (*p* = 0.114). **(E)** Fibers were prepared and stained from cells treated as in **(C)**. The lengths of all fibers in a field, and the total number of Dig-TMP signals on fibers, were determined and used for the calculation of the number of Dig-TMP signals per 10^3^ kb of fiber. The number of Dig-TMP signals scored and the total number of fibers examined were: at 5 μM- 44 and 78; 70 and 98; 54 and 65; at 20 μM- 97 and 69; 81 and 62; 80 and 61 (*N* = 245, 188). The *t*-test showed significant difference (*p* < 0.001) between the two means (2.69, 4.48, *SD* 0.095, 0.285).

In order to develop an approach for imaging psoralen ICLs we had to attach a reliable and effective detection tag. In the first attempt, TMP was linked to rhodamine in an effort to generate a compound whose removal by repair could be visualized directly in live cells over time. Unfortunately, rhodamine is readily taken into mitochondria and little or no signal was observed in the nucleus. Another fluor, Oregon Green, was coupled to TMP. Although the Oregon Green compound did appear in the nucleus it was quickly bleached. Biotin psoralen conjugates have been used for many years to label DNA *in vitro*. However, biotin is a cofactor for carboxylases, with a strong cytoplasmic and mitochondrial presence. Like the rhodamine derivative very little of the biotin-TMP went to the nucleus. Finally, we synthesized the digoxigenin derivative (**Figure 1A**) (Thazhathveetil et al., 2007). Digoxigenin is a frequently used immuno-tag and commercial antibodies against it are available. Cells treated with the Dig-TMP and UVA and immunostained against the Dig tag displayed nuclear fluorescence. Therefore, Dig-TMP was used in all subsequent experiments.

An approach based on imaging a detection tag linked to psoralen adducts cannot distinguish the different products formed by the reaction of the compound with the genomic DNA. Since the goal of our studies was an understanding of how cells respond to ICLs it was of obvious importance to analyze the relative abundance of ICLs and single strand adducts (monoadducts) in genomic DNA following treatment of live cells with TMP/UVA, or Dig-TMP/UVA. DNA was purified from treated cells and subjected to nuclease P1 digestion and HPLC-tandem mass spectrometry. The results indicated that cell treatment with both TMP and Dig-TMP yielded ICL/monoadduct ratios of 10:1. That is, of 11 adducts, 10 were ICLs. Thus it was possible to interpret signals from the tagged psoralen as primarily derived from ICLs (see Huang et al., 2013 for detailed results of this analysis).

### Immunofluorescence of Dig-TMP

Analysis of the Dig-TMP in cells following exposure to the UVA lamp revealed nuclear staining with the apparent exclusion from nucleoli (**Figure 1B**). This staining pattern did not permit any conclusion regarding the density or distribution of Dig-TMP ICls in genomic DNA. In order to address this question we took advantage of imaging technology based on DNA fibers. This approach is usually employed to address questions related to DNA replication. The methodology exploits the incorporation into DNA, during S phase, of nucleoside analogs that can be detected by IF using appropriate antibodies (for the halogenated analogs CldU, IdU, or BrdU) or by chemical conjugation with biotin (to EdU using click chemistry). The IF analyses illuminate tracks on individual DNA fibers stretched out on a glass surface (Schwab and Niedzwiedz, 2011).

Cells were incubated in medium containing CldU for 24 h to uniformly label DNA. They were then treated with 5 μM Dig-TMP and UVA after which DNA fibers were spread on microscope slides and the CldU imaged by conventional IF. Individual Dig-TMP molecules covalently attached to the DNA were detected by immuno-quantum dot technology (Kad et al., 2010; Huang et al., 2013). A field with DNA fibers, some of which have Dig-TMP signals, is shown in **Figure 1C**. Analysis of the fibers indicated that about 10–15% had a Dig-TMP signal on them. The experiment was repeated with a higher concentration of Dig-TMP. Although the frequency of fibers with Dig-TMP signals increased slightly, the number of signals within fibers was clearly enhanced, such that the average distance between them was reduced (**Figures 1D,E**). Psoralen has been used as a probe of chromatin structure and DNA conformation (Wieshahn et al., 1977; Kouzine et al., 2013). We interpret our results as indicating that some regions of the genome are more accessible to the Dig-TMP than others, consistent with prior reports, and, at higher concentrations of compound, those regions are more extensively modified. However, it should be noted that we did not see the appearance of intense localized signals at the higher concentration, suggesting that the Dig-TMP molecules do not react at closely spaced hotspots, in contrast to the clustered breaks introduced by high-linear energy transfer (LET) ionizing radiation (Lorat et al., 2015).

### Laser Localized ICLs

**FIGURE 2:**
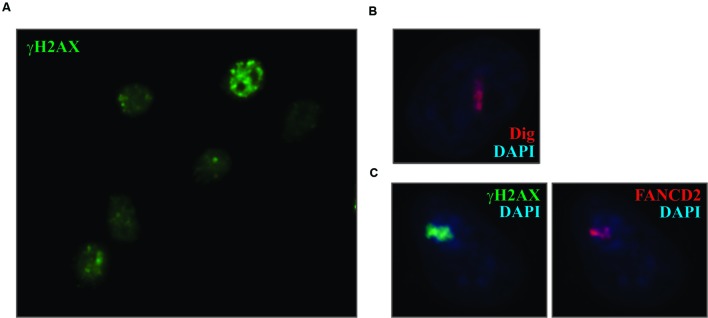
**Laser localized Dig-TMP induces the DDR. (A)** Cells were incubated with 20 μM Dig-TMP and exposed to the UVA lamp. They were fixed and immunostained for γ-H2AX. **(B)** Cells were incubated with Dig-TMP and a defined region of interest was exposed to the 365 nm laser light. Cells were fixed and immunostained for the Dig tag. **(C)** Cells were incubated with Dig-TMP, exposed to the laser in an ROI and after 15 min fixed and immunostained for γ-H2AX (green) and FANCD2 (red).

As shown above, treatment of cells with psoralen/UVA resulted in the introduction of ICLs throughout the nucleus. However, unlike the situation with DSBs, discrete foci of DDR proteins, such as γ-H2AX, did not form (**Figure 2A**). In this regard the treated cells are much like those exposed to UVC. So in order to “create” foci why not just employ the strategy of masking with a micropore filter, as was done successfully with UVC? Unfortunately, while the filters block shortwave UV, they do not block the long wave UV required for psoralen photoactivation. We attempted to construct effective filters by carbon or gold shadowing but were unsuccessful. Consequently, another approach was required.

In order to generate “foci” of ICLs and responding proteins, we employed laser localization. Cells were grown on a glass bottomed dish onto which a cross was marked with a diamond pen. The cells were incubated with Dig-TMP and ROI were exposed to 365 nm laser light. This produced a stripe of localized Dig-TMP adducts which could be displayed by IF against the Dig tag (**Figure 2B**) (Thazhathveetil et al., 2007). The location of individual treated cells was referenced to the cross mark.

The laser localized ICLs were inducers of the DDR, demonstrated by the accumulation in the stripes of γ-H2AX and the Fanconi Anemia protein FANCD2 (Yan et al., 2012) (**Figure 2C**). We have used this approach to detail the recruitment of many DDR and repair proteins to the ICLs (Muniandy et al., 2009; Xu et al., 2010; Yan et al., 2010, 2012; McNeill et al., 2013; Singh et al., 2013; Jiang et al., 2015; Tian et al., 2015). This requires the combination of both compound and light. In the absence of psoralen, exposure to the laser at the intensity used in these experiments does not induce the recruitment of the DDR and repair proteins.

### Distribution of Laser Localized ICLs in Genomic DNA

**FIGURE 3:**
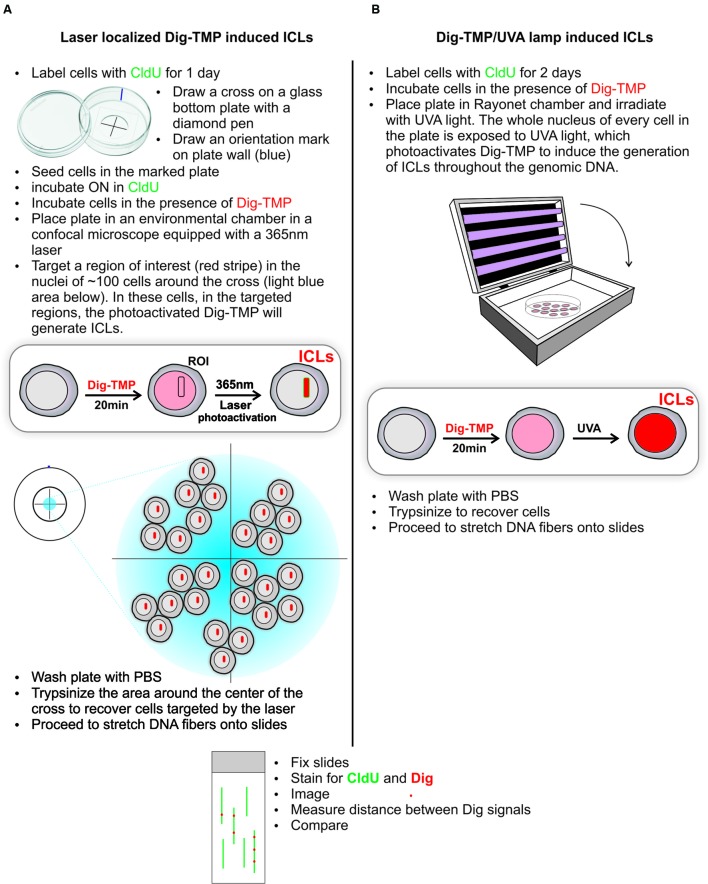
**Distribution of UVA lamp induced and laser localized ICLs**. The experimental design is presented in the schematic. The ICLs, indicated in red, are localized to stripes after laser photoactivation of Dig-TMP at ROIs **(A)**, and distributed throughout the nucleus after exposure of cells to Dig-TMP and a UVA lamp **(B)**.

**FIGURE 4:**
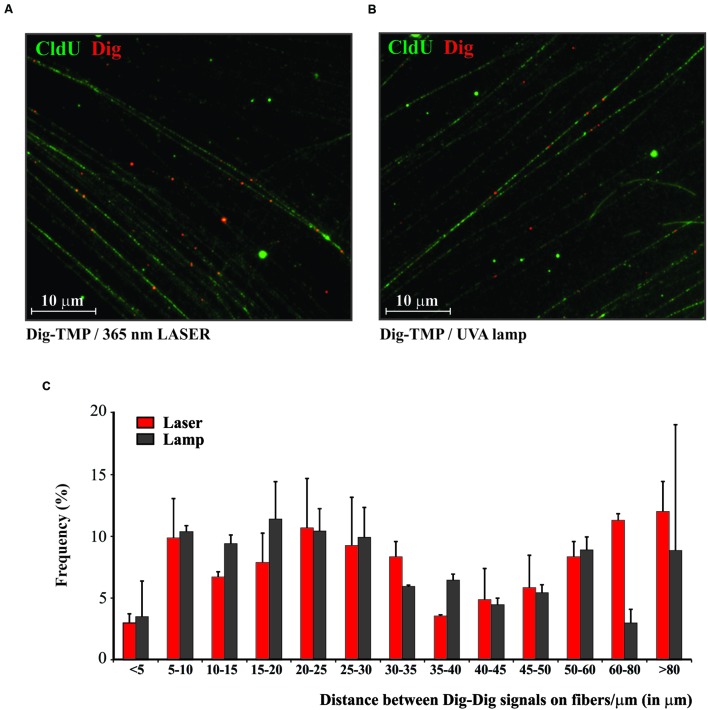
**The distribution of Dig-TMP ICLs on DNA molecules after photoactivation by laser or UVA lamp. (A)** Cells were prelabeled with CldU, seeded in Mattek plates marked with a cross on the growth surface of the glass. The next day cells were incubated in 20 μM Dig-TMP and the ROI in nuclei in cells close to the cross were photoactivated with the laser. Cells in the vicinity of the cross were harvested and placed on a slide and fibers spread, fixed, and immunostained for CldU (green) and Dig-TMP (red). **(B)** Cells were prelabeled with CldU, incubated with 20 μM Dig-TMP and exposed to the UVA lamp. **(C)** The distribution of Dig-TMP signals on fibers from cells with laser or lamp photoactivation. The plot represents results from 3 independent experiments (number of laser localized Dig-TMP signals used for distance measurements in the individual experiments-111, 85, 90; number of Dig-TMP signals induced by UVA lamp used for the measurements-102, 101, 102).

The laser and the UVA lamp are quite different light sources for psoralen photoactivation. Since both are used in experiments to introduce psoralen ICLs it was important to ask if the distribution of laser localized Dig-TMP ICLs in genomic DNA differed markedly from that in experiments with the UVA lamps. Our approach to answering these questions is diagrammed in **Figure 3**. Cells were incubated with CldU for 24 h to uniformly label DNA. They were then incubated in 20 μM Dig-TMP and laser localized ICLs were introduced into ROI in nuclei in cells located close to the cross. Those cells were harvested and DNA fibers were prepared (**Figure 3A**). In parallel, fibers were spread from cells incubated with the same concentration of Dig-TMP and exposed to the UVA lamps (**Figure 3B**). The Dig-TMP adducts were displayed by the immuno-quantum dot technique (**Figures 4A,B**). The distribution of Dig-TMP signals on individual fibers from both preparations was determined (**Figure 4C**). These measurements demonstrated very little difference in Dig-TMP adduct spacing between the two samples. This argued that the distribution of TMP adducts was based on the properties of the psoralen, not the light source.

### Replication Fork Encounters with Laser Localized ICLs

In previous work, we combined DNA fiber technology and immuno-quantum dot imaging in a study of replication fork encounters with Dig-TMP ICLs introduced by exposure to a UVA lamp (Huang et al., 2013). We observed patterns in which replication tracts terminated at ICLs (single sided), as well as those in which replication occurred on both sides of an ICL (double sided). It was of interest to determine the relevance of this observation to events in the stripes containing the laser localized ICLs. Given the obvious differences in light source the immediate question was whether or not there would be replication on fibers containing ICLs induced by the laser.

**FIGURE 5:**
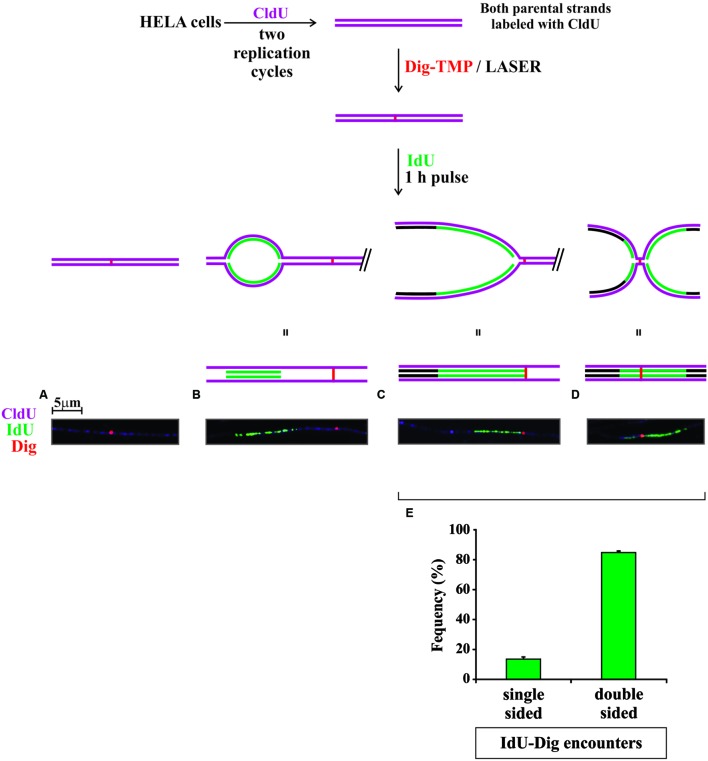
**Replication in the vicinity of laser localized Dig-TMP ICLs**. Cells were seeded on plates marked with a cross, incubated with 20 μM Dig-TMP, and ICLs introduced by laser photoactivation in ROI in nuclei in cells close to the cross. The cells were then incubated at 37°C for 1 h in the presence of 10 μM IdU. Cells near the cross were recovered, placed on a slide and fibers spread, and stained as before for CldU (purple), IdU (green), and Dig-TMP (red). Four kinds of fibers were scored: **(A)** those containing a Dig-TMP without a replication tract; **(B)** those with a replication tract and, at some distance, a Dig-TMP; **(C)** those in which replication had occurred immediately adjacent to one side of the Dig-TMP; and **(D)** those on which replication tracts were on both sides of the Dig-TMP. **(E)** Quantitation of the relative frequencies of fibers with replication on one or both sides of the Dig-TMP. Results from three independent experiments are represented (numbers of Dig-TMP signals with: replication on one side- replication on both sides; 9-55; 7-42; 3-21). The *z*-test showed significant difference (*p* < 0.001) between the two proportions (averaged proportions 0.138, 0.862, *N* = 137).

The experimental approach is diagrammed in **Figure 5**. Cells were incubated with CldU for 24 h to prelabel cellular DNA. The medium was changed, and they were incubated with Dig-TMP. Then ROI in nuclei in cells close to the cross were exposed to the laser. The medium was changed to one containing IdU and the cells returned to the incubator for 1 h. Cells proximal to the cross were harvested as before (**Figure 3A**), DNA fibers spread, and the CldU prelabel, the IdU replication label, and the Dig-TMP displayed. The prelabel made it possible to trace the continuity of fibers over much greater length than possible if only replication tracts had been displayed. We observed fibers with Dig-TMP spots, but no replication tracts on the fiber (**Figure 5A**). Replication tracts were observed on fibers at various distances from Dig-TMP signals (**Figure 5B**), indicating that there was indeed replication on fibers isolated from the ROI exposed to the laser. In addition there were tracts terminated by Dig-TMP spots, as well as those with embedded Dig-TMP signals (**Figures 5C,D**). The relative frequencies of each, 15% single sided, 85% double sided (**Figure 5E**), were very similar to what had been observed previously in experiments in which photoactivation was with the lamp (Huang et al., 2013). These results indicated that replication occurred in similar fashion in the vicinity of Dig-TMP ICLs regardless of the UVA source.

We conclude from these experiments that the fundamentals regarding psoralen ICL formation driven by the UVA lamp and laser are quite similar such that results from the one approach can inform the other.

## Discussion

Laser induced DNA damage is the basis of a substantial literature on the induction of the DDR and the repair of DSBs as monitored by surrogate markers. As noted above, the difficulty of direct identification of breaks precludes assessment of lesion frequency and spacing in the ROI. Furthermore, the possibility of extensive damage due to exposure of DNA to the high intensities associated with lasers has been a source of concern (Williams et al., 2007). In this communication, we have resolved these issues in regard to the psoralen ICLs. Our experiments indicate that the density of psoralen DNA photoproducts is similar regardless of the light source for photoactivation. Consequently, it is reasonable to extrapolate results and conclusions from experiments with one UVA source to experiments with the other. Furthermore, the demonstration of replication tracts in the immediate vicinity of the psoralen adducts argues that replication fork encounters with laser localized ICLs do occur, with a pattern distribution quite like that observed with UVA lamp induced psoralen ICLs. These observations enable experiments designed to examine replication fork dynamics in the vicinity of the laser activated psoralen ICLs. Such experiments are not possible with laser induced DSBs, because there is no methodology for direct detection of the breaks.

The laser localization strategy obviously requires photo-dependent adduct formation, and so is limited to appropriate compounds. However, display of DNA damage on DNA fibers requires only a DNA adduct detectable by chemical conjugation or immunological reagents. For example, a DNA reactive agent linked to one of the partners in azide/alkyne click chemistry would be applicable. Similarly, antibodies or specific binding proteins against DNA adducts (carcinogens, UV photoproducts) could also be employed (Poirier, 1993; Dreze et al., 2014; McCready, 2014). Thus, we think it likely that the fiber strategies can be generalized to other DNA damaging agents, and could be used to address question regarding adduct distribution, repair, and replication fork encounters.

## Author Contributions

JH, HG, MP, PM, JG, MAB, and MMS participated in designing and performing experiments, analyzing results and contributed to generating the figures and writing the paper.

## Conflict of Interest Statement

The authors declare that the research was conducted in the absence of any commercial or financial relationships that could be construed as a potential conflict of interest.
